# Understanding how non-coding genomic polymorphisms affect gene expression

**DOI:** 10.1038/mp.2015.226

**Published:** 2016-01-26

**Authors:** S E Koester, T R Insel

**Affiliations:** 1 Division of Neuroscience and Basic Behavioral Science, NIMH, NIH, Bethesda, MD, USA

## Abstract

The NIH Common Fund GTEx project is designed to serve as a data and post-mortem tissue resource to the research community. The project is testing the role of genomic variation in altering gene expression across a wide array of tissues in a large number of human post-mortem donors. Both data and tissue samples are available to the research community for additional studies.

The publication of the results from the Psychiatric GWAS Consortium showing 108 genetic loci linked to a diagnosis of schizophrenia^1^ represents a milestone for psychiatric genetics but also presents a challenge. How will we assess these loci and find which are linked to brain changes that may be amenable to intervention? Similar large-scale genomics efforts in other major mental disorders are raising the same issues. With the Genotype Tissue Expression (GTEx) Project, the NIH Common Fund seeks to bridge the gap between SNPs of unknown function and changes in gene expression in human brain and many other organs and tissues.

The key question is how significant GWAS loci alter biological processes resulting in risk for or protection from disease. Answering this question requires access to the disease tissues of interest. However, in many illnesses, including psychiatric disorders, the lack of access to primary disease tissues, such as brain, presents a challenge. The GTEx project was designed to address this gap by collecting many different tissue types from the same post-mortem adult donors with broad inclusion criteria and rigorous quality management.^2^ The goal of the GTEx project is to create a data and tissue resource to serve as a reference set for studies of the genomic basis of disease. Over 900 post-mortem human donors have been recruited and tissues collected through Organ Procurement Organizations and Tissue transplant collection sites, following collection of transplantable organs and tissues. Functional impact in tissues from a variety of organs, including 12 distinct brain regions, has been assessed by Next Generation RNA sequencing (RNAseq). In addition, Whole Genome and Whole Exome Sequences (WGS, WES) were generated from blood from each donor.

Other studies have measured gene expression levels in brain (for example, BrainSpan, http://www.brainspan.org/; BrainCloud;^3^ http://braincloud.jhmi.edu/), but GTEx is the first project to expand the analysis to a broad array of both brain and non-brain tissues from a large number of individuals. These large numbers allow the project to measure the extent to which changes in the genome affect changes in gene expression throughout the human body. In studies like GTEx transcription is treated statistically as a quantitative trait, so genomic loci that correlate with altered gene expression are expression quantitative trait loci (eQTLs). Figure 1 shows a hypothetical example of a tissue-specific eQTL and its effect on transcription. The addition of expression data in multiple tissues from the same donor enhances the resource, both computationally (that is, by increasing the power to compute eQTLs in individual tissues) and comparatively. The data will ultimately allow researchers to follow-up on results from GWAS studies, to learn whether/which GWAS significant variants are correlated with and may result in changes in gene expression in a tissue-specific manner or across multiple tissues.

The researchers funded by the GTEx project have organized a consortium for joint analyses of the GTEx data. Members of the GTEx Consortium are developing and applying new methods to find QTLs that go well beyond gene expression, examining effects on protein truncating variants, protein expression, imprinting, and many others. The value of the GTEx data as a reference data set is only starting to be realized.

Analyses of the first major data release were published earlier this year. These data included transcriptional data from a set of 9 tissues from 175 donors, allowing calculation of the first *cis*-eQTLs in these tissues.^4^ Data are released regularly; the most recent release (www.gtexportal.org, dbGaP accession phs000424.v6.p1; October (2015)) includes sufficient cases with both WGS and RNAseq from donors including brain samples to allow calculation of the first brain eQTLs of the GTEx project. The Consortium analyses will be tremendously useful applied to the newly available brain data. In particular, sophisticated models for joint analysis of tissues enabled the discovery of nearly three times as many eQTLs as found in single tissues. While these initial results were focused on non-brain tissues, we might expect to see similar increases in the discovery of eQTLs in brain.

Another recent Consortium manuscript^5^ examines gene expression levels across many tissues and the entire transcriptome. The paper provides significant new data about differences in expression rates across tissues including brain. In particular, the authors show that expression signatures of different tissues are frequently dominated by a small set of genes that vary from tissue to tissue. In brain tissues, as in other high-metabolizing tissues, these signatures are dominated by a subset of mitochondrial genes.

While the GTEx project was designed to compute eQTLs, additional analyses are already illustrating the breadth of opportunity offered by the data set. Researchers are able to mine the data based on specific disorders or alleles of interest to look for effects on transcription across multiple tissue types. GTEx researchers are also discovering loci correlated with tissue-specific differential splicing, or splicing QTLs (sQTLs). GTEx Consortium researchers are also currently investigating molecular effects beyond messenger RNA transcription. These include studies of DNA and RNA methylation, protein levels, DNAse1 hypersensitivity, and somatic mutation analysis assays in a subset of the tissues collected. Shared histopathology images of the tissue samples will soon allow studies of effects of alleles or expression levels on anatomy, both for brain and other organs and tissues. In addition, brain (frozen) and peripheral tissue (fixed, including paraffin embedded) from many donors (www.gtexportal.org) are banked, consented for broad research use, and may be requested (http://www.gtexportal.org/home/samplesPage) to support further studies of these donors.

Individual GWAS hits for complex disorders have generally had low or modest effects on observable phenotypes, such as behavior. However, new computational methods such as PrediXcan^6^ are capitalizing on the wealth of data generated by GTEx along with information from GWAS to glean a deeper understanding of how individual genomic differences alter gene expression. GTEx surveyed gene expression in a heterogeneous mix of cell types in each sample, possibly obscuring larger effects in specific cell types. However, a collaboration between GTEx and the Common Fund Single Cell Analysis Program will generate data sets to support methods to computationally deconvolute gene expression signatures of individual cell types in brain and other tissues. In addition, Allen Institute for Brain Studies is developing a relevant atlas of transcriptional signatures of brain cell types (http://celltypes.brain-map.org/). The combination of these signatures of individual cell types with the depth of the large number of donors to GTEx will enable a remarkable understanding of the variability of cell types and expression patterns across brain regions and individuals.

In closing, we acknowledge the generous gift of post-mortem tissue from the GTEx donors and their family decision makers that makes this research possible. With the tremendous opportunity presented by this rich resource comes great responsibility. As one of the GTEx donor family members put it: ‘Just remember that I gave you something that means more than words could ever describe to anybody.... It is something that will help everybody, for the greater good.'

## Figures and Tables

**Figure 1:**
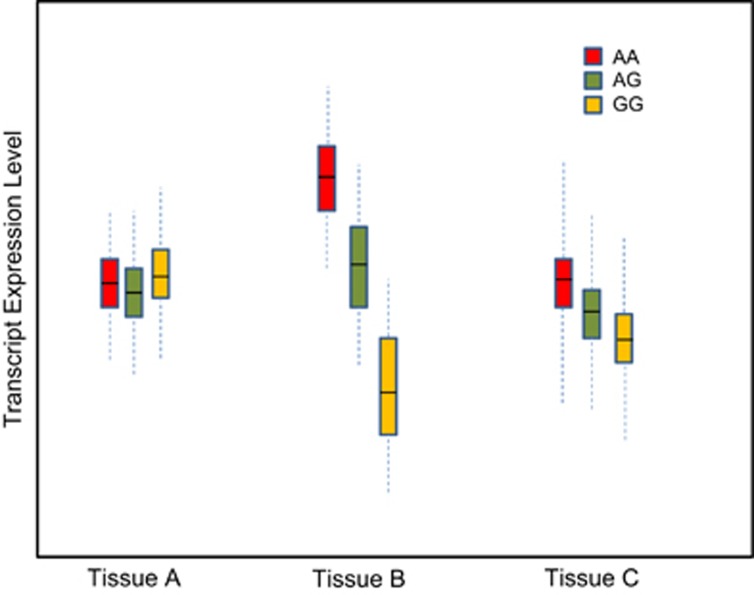
Hypothetical eQTL demonstrated through differential expression across three tissues. Each colored bar represents expression level under the different SNP alleles at a specific locus. In Tissue A, the SNP locus does not correlate with a significant change in gene expression. In Tissue B and C the variants at the locus correlate with a strong or small change in expression, respectively.
